# Supplementary material for: The interactome of intact mitochondria by cross-linking mass spectrometry provides evidence for coexisting respiratory supercomplexes
Source: Mol Cell Proteomics. 2017 Dec 8;17(2):216–32. doi: 10.1074/mcp.RA117.000470 (PMC5795388; doi:10.1074/mcp.RA117.000470)
Supplement: Supplemental Data [file supp_RA117.000470_133922_0_supp_23978_fzffwf.zip › spectra_annotation/mito_spectra_annotation/173-1-6-1-3-1.pdf]

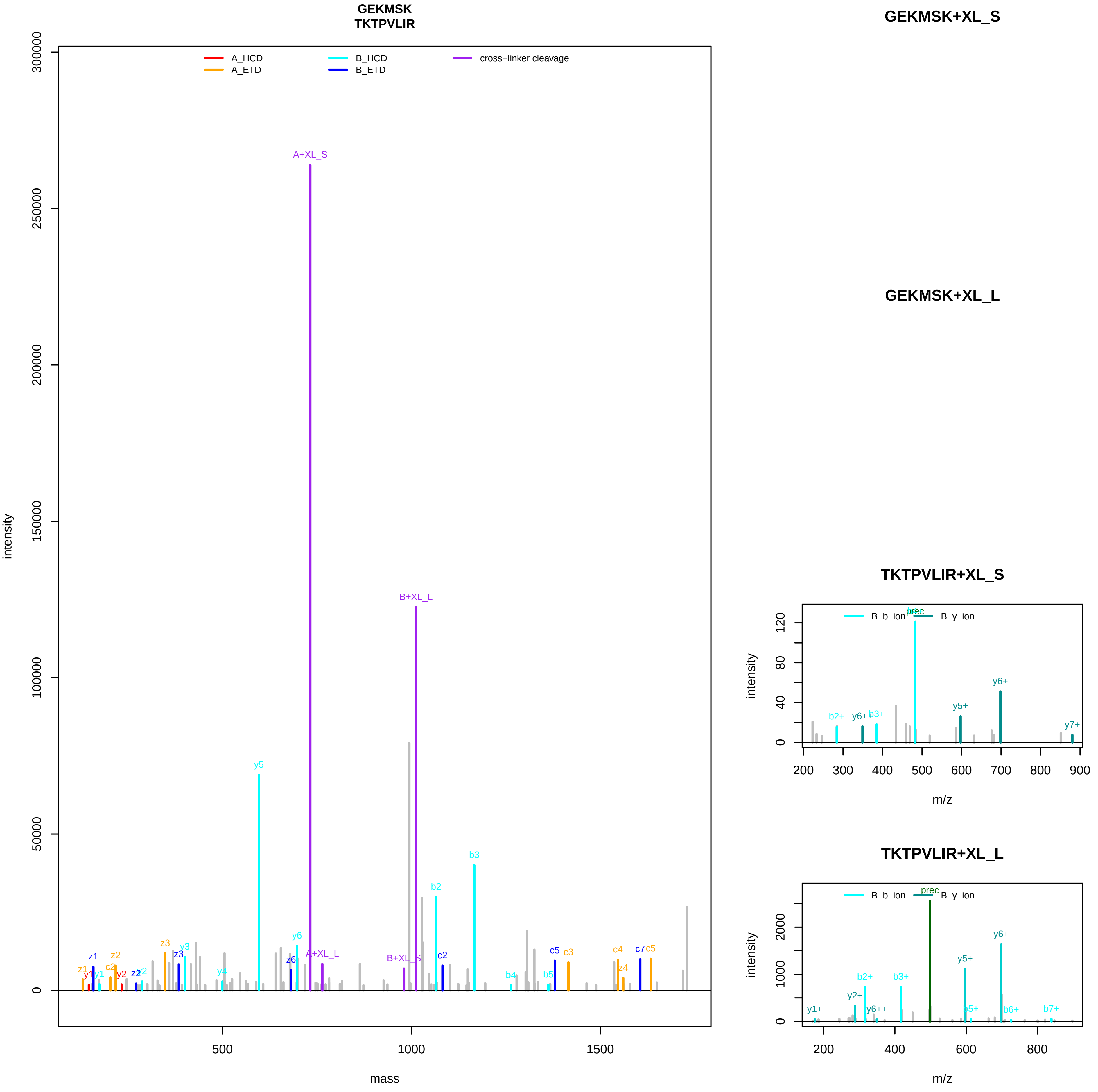
GEKMSK
TKTPVLIR
A_HCD
A_ETD
B_HCD
B_ETD
cross−linker cleavage
intensity
mass
0
50000
100000
150000
200000
250000
300000
500
1000
1500
A+XL_S
B+XL_L
A+XL_L
B+XL_S
y5
y6
b2
b3
b4
b5
c2
c3
c4
c5
c7
z1
z2
z3
z4
z6
y1
y2
y3
y4
c2
z2
z3
y2
z1
GEKMSK+XL_S
GEKMSK+XL_L
TKTPVLIR+XL_S
B_b_ion
B_y_ion
prec
b2+
b3+
y5+
y6+
y6++
y7+
m/z
0
40
80
120
200
300
400
500
600
700
800
900
TKTPVLIR+XL_L
y1+
y2+
b2+
b3+
y5+
y6+
y6++
b5+
b6+
b7+
0
1000
2000
200
400
600
800
